# Engineering Attenuated Virulence of a *Theileria annulata*–Infected Macrophage

**DOI:** 10.1371/journal.pntd.0003183

**Published:** 2014-11-06

**Authors:** Nadia Echebli, Moez Mhadhbi, Marie Chaussepied, Catherine Vayssettes, James P. Di Santo, Mohamed Aziz Darghouth, Gordon Langsley

**Affiliations:** 1 Laboratoire de Biologie Cellulaire Comparative des Apicomplexes, Faculté de Médicine, Université Paris Descartes - Sorbonne Paris Cité, Paris, France; 2 Inserm U1016, Cnrs UMR8104, Cochin Institute, Paris, France; 3 Laboratoire de Parasitologie, Ecole Nationale de Médecine Vétérinaire, Sidi Thabet, Tunisia; 4 Innate Immunity Unit, Department of Immunology, Pasteur Institute, Paris, France; 5 Inserm U688, Pasteur Institute, Paris, France; 6 Institution de la Recherche et de l'Enseignement Supérieur Agricoles, Tunis, Tunisia; Barcelona Centre for International Health Research (CRESIB) and Institució Catalana de Recerca i Estudis Avançats (ICREA), Spain

## Abstract

Live attenuated vaccines are used to combat tropical theileriosis in North Africa, the Middle East, India, and China. The attenuation process is empirical and occurs only after many months, sometimes years, of *in vitro* culture of virulent clinical isolates. During this extensive culturing, attenuated lines lose their vaccine potential. To circumvent this we engineered the rapid ablation of the host cell transcription factor c-Jun, and within only 3 weeks the line engineered for loss of c-Jun activation displayed *in vitro* correlates of attenuation such as loss of adhesion, reduced MMP9 gelatinase activity, and diminished capacity to traverse Matrigel. Specific ablation of a single infected host cell virulence trait (c-Jun) induced a complete failure of *Theileria annulata*–transformed macrophages to disseminate, whereas virulent macrophages disseminated to the kidneys, spleen, and lungs of Rag2/γC mice. Thus, in this heterologous mouse model loss of c-Jun expression led to ablation of dissemination of *T. annulata*–infected and transformed macrophages. The generation of *Theileria*-infected macrophages genetically engineered for ablation of a specific host cell virulence trait now makes possible experimental vaccination of calves to address how loss of macrophage dissemination impacts the disease pathology of tropical theileriosis.

## Author Summary

Tropical theileriosis is a leukaemia-like disease of cattle caused by the Apicomplexa parasite *Theileria annulata*. Live attenuated vaccines are used to control the mortality and morbidity of tropical theileriosis and each endemic country produces its own vaccine by isolating a virulent clinical isolate and then growing the infected and transformed macrophages for many months in the laboratory. With time, virulence is progressively lost, and loss is evaluated from time to time by injecting infected macrophages into calves and monitoring any resulting pathology. If the calves demonstrate clinical symptoms, *in vitro* culture of vaccine lines continues, and eventually attenuation is achieved after approximately two years. The process is empirical, and with time in culture the infected macrophage lines lose their vaccine potential such that when cattle are injected the immune response to the vaccine can be compromised. To circumvent these problems we engineered the rapid loss of a host macrophage virulence trait and obtained complete attenuation of dissemination of *T. annulata*–transformed macrophages when tested in an immune-compromised mouse model. This rational, rather than empirical, approach now allows injecting into calves *Theileria*-infected macrophages engineered for attenuation to evaluate the role of *Theileria*-infected macrophage dissemination in the pathology of tropical theileriosis.

## Introduction

Tropical theileriosis caused by *Theileria annulata* is a major parasitic disease of cattle and is endemic in North Africa, the Mediterranean basin and Asia (India and China). Attenuated live vaccines against tropical theileriosis have been used with success in endemic countries, in spite of MHC differences between the different live vaccines and vaccinated animals [1]. One of the earliest molecular events associated with attenuation of infected macrophages was loss in Activator Protein-1 (AP-1)-driven *mmp9* expression [2], [3]. Concomitant with attenuation there is an alteration in the composition of the different AP-1 family members binding to the *mmp9* promoter [4]. AP-1 is a mixture of dimers made up of members of the Jun family (c-Jun, JunB, and JunD), associated with proteins of the Fos (c-Fos, FosB, Fra1, and Fra2), and ATF2 families [5]. Thus, the outcome of AP-1 activation results from combinatorial interactions between different family members binding to the promoters of AP-1-target genes. C-Jun N-terminal Kinase (JNK) and ATF2 are constitutively activated in both *T. annulata* and *T. parva* infected leukocytes [6], [7], [8], [9]. Constitutive JNK activation promotes survival, proliferation and metastasis of *Theileria*-infected leukocytes [10], [11], [12] indicating that JNK is a major player in *Theileria*-induced transformed leukocyte virulence via its phosphorylation of c-Jun at residues Ser63 and Ser73 that increases AP-1 transactivation [12].

Matrix Metallo Proteinases (MMPs) and their inhibitors (TIMPs) play significant roles in immunity, inflammation and cancer, for reviews see [13]. A disintegrin and metalloproteinases (ADAMs) are a family of proteins with similarity to snake venom reprolysins, with a metalloproteinase domain present in MMPs. Due to their roles in cell adhesion, migration and membrane protein shedding ADAM expression is often aberrant in tumours, for review [14]. Of particular relevance to *Theileria*-transformed macrophages, where secreted TNF [15] has recently been shown to play an important role infected macrophage motility [16], ADAM19 can function as a TNF “sheddase” releasing membrane bound TNF into the circulation [17], [18]. In spite of its established role as a TNF sheddase and its deregulation in tumour cell lines ADAM19 expression by *Theileria*-infected leukocytes has not yet been described.

No engineered vaccine exists against tropical theileriosis and so we describe here the first engineered attenuated *T. annulata*-infected line via functional inactivation of a host macrophage transcription factor (c-Jun) that we have previously shown mediates *T. parva*-infected B cell metastasis [12]. By rapidly targeting a host cell function we have generated an attenuated macrophage line displaying many of the ablated virulence phenotypes in just 3 weeks, rather than the circa 2 years of continuous culture typical of live attenuated vaccines to tropical theileriosis.

## Materials and Methods

### *Theileria annulata*–infected cell lines

Characterisation of the Jed 4 line first isolated in Tunisia has been reported [19] and in this study virulent (early-passage) Jed 4 corresponds to passage 18. V-Delta 169 is a pool of Jed 4 cells, which were generated by retroviral transduction of Delta 169-c-Jun [12] that over-expression of truncated c-Jun lacking the first 169 amino acids ablates c-Jun-mediated transcription, by sequestering into inactive complexes endogenous c-Jun and c-Jun binding partners [20].

The retroviral transduction introduces the vector into 80% of target cells and we performed 2 successive rounds of transduction and moreover, the vector encodes resistance to G418 and all transduced macrophages were drug-resistant. 100% of cells in the line therefore, expressed the dominant negative c-Jun construct that is stably integrated into the macrophage genome.

All cultures were maintained at 37°C with 5% CO_2_ in RPMI-1640 medium supplemented with 10% Foetal Bovine Serum (FBS). Cultures were passaged 24 h before harvesting to maintain the cells in an exponential growth phase.

V: Jed4p18 = virulent line

V-Delta 169 = engineered attenuated line

### Transient transfection experiments and luciferase assays

Transient transfection experiments were carried out using a 3*TRE (Tissue Responsive Element) coll-luciferase reporter plasmid (2 µg; [9], pGL2luc basic (2 µg), as per [9]. Cytomegalovirus-driven β-galactosidase-expressing vector (500 ng) was included in each sample for standardization of transfection efficiency. For luciferase assays, cells were harvested 48 h after transfection and luciferase activity was assayed in a microplate luminometer (Berthold technologies, Centro LB960) using a luciferase assay reagent (Dual-Light Chemiluminescent Reporter Gene Assay System for the combined detection of luciferase and β-galactosidase, Applied Biosystems). Luciferase activity was compared with the basal levels obtained for pGL2-transfected cells.

### Western blot analysis

Cells were harvested and extracted with lysis buffer (Hepes 20 mM, pH 8; NaCl 150 mM; EDTA 2 mM; Nonidet P40 1%; SDS 0.1%; sodium deoxycholate 0.5% containing protease inhibitors (Complete mini EDTA free, Roche) and phosphatase (PhosSTOP, Roche). Lysates were centrifuged at 13,000 rpm for 15 min at 4°C, and supernatants collected. Equal amounts of protein were separated by SDS-PAGE, transferred to nitrocellulose membrane (Protran, Whatman) at 30 V overnight at 4°C and blocked with 4% skimmed milk for 2 h. The antibodies used in immunoblotting were as follows: Anti-pATF2 (pThr71) (sc-7982-R, Santa Cruz Biotechnology, Santa Cruz, CA); anti-ATF2 (sc-6233, Santa Cruz Biotechnology, Santa Cruz, CA); anti-c-Jun (sc1694, Santa Cruz Biotechnology, Santa Cruz, CA); anti-MMP9 (AV33090; Sigma); anti-ADAM19 (ARP49780_P050. Aviva Systems Biology); anti-Actin (I 19-sc1616, Santa Cruz Biotechnology, Santa Cruz, CA).

### Total RNA extraction

Total RNA was isolated from *T. annulata*-infected Jed 4 macrophages using the RNeasy mini kit (Qiagen) according to the manufacturer's instructions. The quality and quantity of the RNA was determined using a Nanodrop spectrophotometer and gel electrophoresis. mRNA was reverse transcribed to first-strand cDNA and the relative level of each transcript was quantified by real-time PCR using SYBR Green detection. The detection of a single product was verified by dissociation curve analysis and relative quantities of mRNA calculated using the method described by [21]. Relative amount of *hprt1* was used to normalise mRNA levels. Primer sequences used are as follows: ICAM-1: Forward 5′-GCAACTTCTCCTGCTCTGCT-3′, Reverse 5′- CTCCAGGGTCTGGGTTTTGT-3′; CD49: Forward 5′- AGCCCCTCAACATGAACAGA-3′, Reverse 5′- TCCCACGAGTAGGTCTGAGA-3′; ITGB5: Forward 5′- TGGAACTTGGCGAACTCTCT-3′, Reverse 5′- GGGTTTGCACTTCTGGTCAT-3′; CD69: Forward 5′- AATGGTCAAATGGCCAAGAA-3′, Reverse 5′- TCTCAGACCCCGTAAGGTTG-3′; TIMP3: Forward 5′- ACAGGCCGAGTCTATGATGG-3′, Reverse 5′- ACAGCCCAGGTGATATCGATAGTT-3′; ADAM19: Forward 5′- GGAAGGACATGAATGGGAAA-3′, Reverse 5′- CTCGGAACTCTGACACTGGA-3′; MMP9: Forward-5′ CCCATTAGCACGCACGACAT-3′, Reverse 5′- TCACGTAGCCCACATAGTCCA-3′; HRPT1: Forward-5′ TGGACAGGACCGAACGGCT-3′, Reverse 5′- TAATCCAACAGGTCGGCAAAG-3′.

### Adhesion assay

A 96-well plate was coated with bovine fibronectin (Sigma #F1141), 2 µg/cm^2^ diluted in double distilled water overnight at 4°C. Then washed twice with 100 µL 0.1% BSA in

RPMI-1640 and blocked for 1 h at 37°C by 0.5% BSA dissolved in RPMI-1640. After two washes, 1×10^4^ cells were added to each well and incubated at 37°C, 5% CO_2_ for 30 min. Non-adherent cells were removed by washing the wells three times before fixing with 100 µL 4% paraformaldehyde for 10 min at room temperature (RT). Following one further wash, wells were stained with 100 µL of crystal violet (1 mg/ml) for 10 min at RT. Wells were extensively washed with distilled water and air-dried. Samples were re-suspended by 30 min incubation at RT in 100 µL 2% SDS, 2% ethanol before reading the optical density at 595 nm.

### *In vitro* invasion assays

The invasive capacity of Jed 4 macrophages was assessed *in vitro* using matrigel migration chambers and the culture coat 96-well medium BME cell invasion assay kit (Culturex Instructions, 3482-096-K). After 24 h of incubation at 37°C each well of the upper chamber was washed once in buffer. The top chamber was placed on the receiver-plate. 100 µL of cell dissociation solution/Calcein AM were added to the bottom chamber of each well, incubated at 37°C for 1 h to fluorescently label cells and dissociate them from the membrane, before 485 nm excitation and reading the 520 nm emission using the same parameters as the standard curve.

### Zymography

The identification of MMP9 proteinase activity was performed using gelatin zymography by electrophoresis of serum-free conditioned medium collected from confluent cells. 10 ml of medium were loaded under non-denaturing conditions onto polyacrylamide zymogram gels supplemented with 0.1% gelatin to detect the presence of MMP9. Electrophoresis was performed at a constant voltage of 125 V for 90 min in 1× Tris-Glycine SDS. Gels were washed in renaturing buffer and placed overnight in incubation buffer, stained with Coomassie brilliant blue R-250 (Sigma, Poole, Dorset, U.K.) and destained with gel-clear destain solution (250 mg Coomassie Brilliant Blue G-250 (Sigma B-1131)+125 mL methanol +50 ml glacial acetic acid +350 ml double-distilled H_2_O). Normally, areas of gelatin degradation appear as transparent bands on the blue background, but for imagery the contrast was inverted to give a black band on a clear background. A set of wide range molecular mass marker (Sigma) was used to estimate molecular mass.

### Dissemination of transformed Jed4 macrophages *in vivo*

The attenuated line, V-Delta 169 and virulent line (V: Jed4p18) macrophages were washed in PBS and injected subcutaneous (1×10^6^ in 0.2 mL of PBS) into Rag2/γC mice [22]. Three independent experiments were performed, such that each infected macrophage type was injected into a total of 36 mice. Each group of 18 mice contained 8 females and 10 males that were sacrificed and organs examined 12 weeks after injection. For detection of disseminated *T. annulata*-transformed Jed 4 macrophages, kidneys, spleens and lungs were dissected.

### Ethics statement

A detailed protocol (number 12–26) entitled “A recombinant vaccine against bovine tropical theileriosis caused by *Theileria annulata*” describing the proposed mice experiments was submitted to and approved (number CEEA34.GL.03312) by the ethics committee for animal experimentation at the University of Paris-Descartes. The university ethics committee is registered with the French National Ethics Committee for Animal Experimentation that itself is registered with the European Ethics Committee for Animal Experimentation. The right to perform the mice experiments was obtained from the French National Service for the Protection of Animal Health and satisfied the animal welfare conditions defined by laws (R214-87 to R214-122 and R215-10) and GL was responsible for all experiment as he holds the French National Animal Experimentation permit with the authorisation number (B-75-1249).

### DNA preparation and PCR amplification of micro-satellite loci

The micro-satellite loci have been described [23], DNA of kidneys, spleens and lungs was purified using a Qiagen QIAamp DNA Mini Kit according to the manufacturer's instructions. Primers were designed to the unique sequence flanking a subset of repeat loci and used to amplify DNA for parasite detection and allele size polymorphism.

Ts4: forward 5′-ATACTGGAGAGTAAGCTAAC-3′

Ts4: reverse 5′CAAGGCCATTCAACTTGACATC-3′

Ts6: forward 5′-CATCCTTTGACCTACTGATTGTAC-3′

Ts6: reverse 5′-CGGTAGTACCAGTTAATACTGTC-3′

Ts8: forward 5′-TAAACGATTAAAATCAAGTG-3′

Ts8: reverse 5′-ATTGGAAATGGTGAAATAATGAG-3′

Ts25: forward 5′-CGCCATCAGTAGTCATCTCAG-3′

Ts25: reverse 5′-GACGACCATAACTGGGAAGTCAAC-3′

DNA preparations from each stock were PCR amplified in a total reaction volume of 50 µL under conditions described previously [24] using the following thermocycler conditions: 94°C for 2 min, 30 cycles of 94°C for 50 s, 50–60°C for 50 s and 65°C for 1 min with a final extension period of 5 min at 65°C. The amplicons were separated on a 2% agarose gels and stained with ethidium bromide. Gels were photographed under ultra-violet transillumination and the size of each PCR product determined by reference to a 200 bp DNA ladder (Eurogentec, MW 1700-02).

## Results

### Suppression of c-Jun leads to down-regulation of AP-1 activity

**Figure 1:**
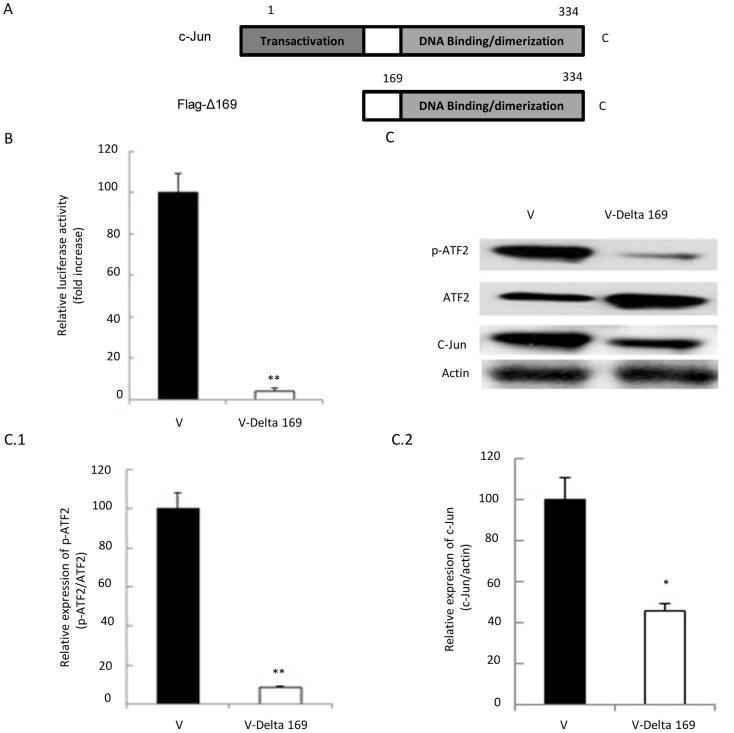
Suppression of c-Jun leads to down-regulation of AP-1 activity. **A**, Flag-tagged dominant-negative mutant of c-Jun, lacking the first 169 amino acid transactivation domain (Flag-Δ169) was introduced into *T. annulata*-infected macrophages. **B**, Suppression of c-Jun reduces AP-1 activity in *T. annulata*-infected macrophages, as measured by 3XTRE-driven luciferase activity (TRE-luc). The activity of AP-1 is 20 times lower in the vaccine line (V-Delta 169) compared to the control line (V). **C**, Western blot analysis of the expression of two different AP-1 family members in vaccine and control lines showing that expression of c-Jun is decreased and ATF2 increased in vaccine line. In spite of an increase in ATF2 protein the level of phospho-ATF2 (p-ATF2) is markedly reduced. Protein amounts were compared to actin and decreases in p-ATF2 and c-Jun quantified using Image J (C1 and C2).

A Flag-tagged dominant-negative mutant of c-Jun, lacking the first 169 amino acid transactivation domain (Flag-Δ169, [25]) was introduced into *T. annulata*-infected macrophages (Figure 1A). AP-1-driven transcription was estimated by co-transfecting a luciferase reporter gene, whose expression is driven by three copies of the AP-1 binding element [26]. Over-expression of Flag-Δ169-c-Jun reduces by 20-fold AP-1-driven luciferase activity in the engineered line (V-Delta 169) compared to the virulent Jed 4 macrophage cell line (V: Jed4p18) that harbours vector only (Fig. 1B). Furthermore, western blot analysis of two AP-1 family members in the virulent and engineered lines showed that the expression of c-Jun and the phosphorylation of ATF2 were decreased (Fig. 1C). The decrease in expression of c-Jun and ATF2 phosphorylation was quantified using Image J (Fig. 1C1 and C2). We have previously shown that ectopic expression of Flag-Δ169-c-Jun in B cells infected with *T. parva* also induces down-regulation of c-Jun [12] and now, we observe that in contrast it induces upregulation in ATF2 protein levels, while phosphorylation of Thr71 in ATF2 is greatly reduced. The down-regulation in phosphorylation of ATF2 is therefore, not due to a decrease in protein levels, but due to Flag-Δ169-c-Jun mediated ablation of Thr71 phosphorylation, likely through reduced transcription of a Thr71-specific kinase. Thus, ectopic expression of Δ169-c-Jun reduces the levels of c-Jun, an established c-Jun target gene [27], without noticeably affecting the proliferation of different types (B cells [12] and macrophages, data not shown) of *Theileria*-transformed leukocytes.

### Role of c-Jun in transformed Jed 4 macrophage adhesion to fibronectin

**Figure 2:**
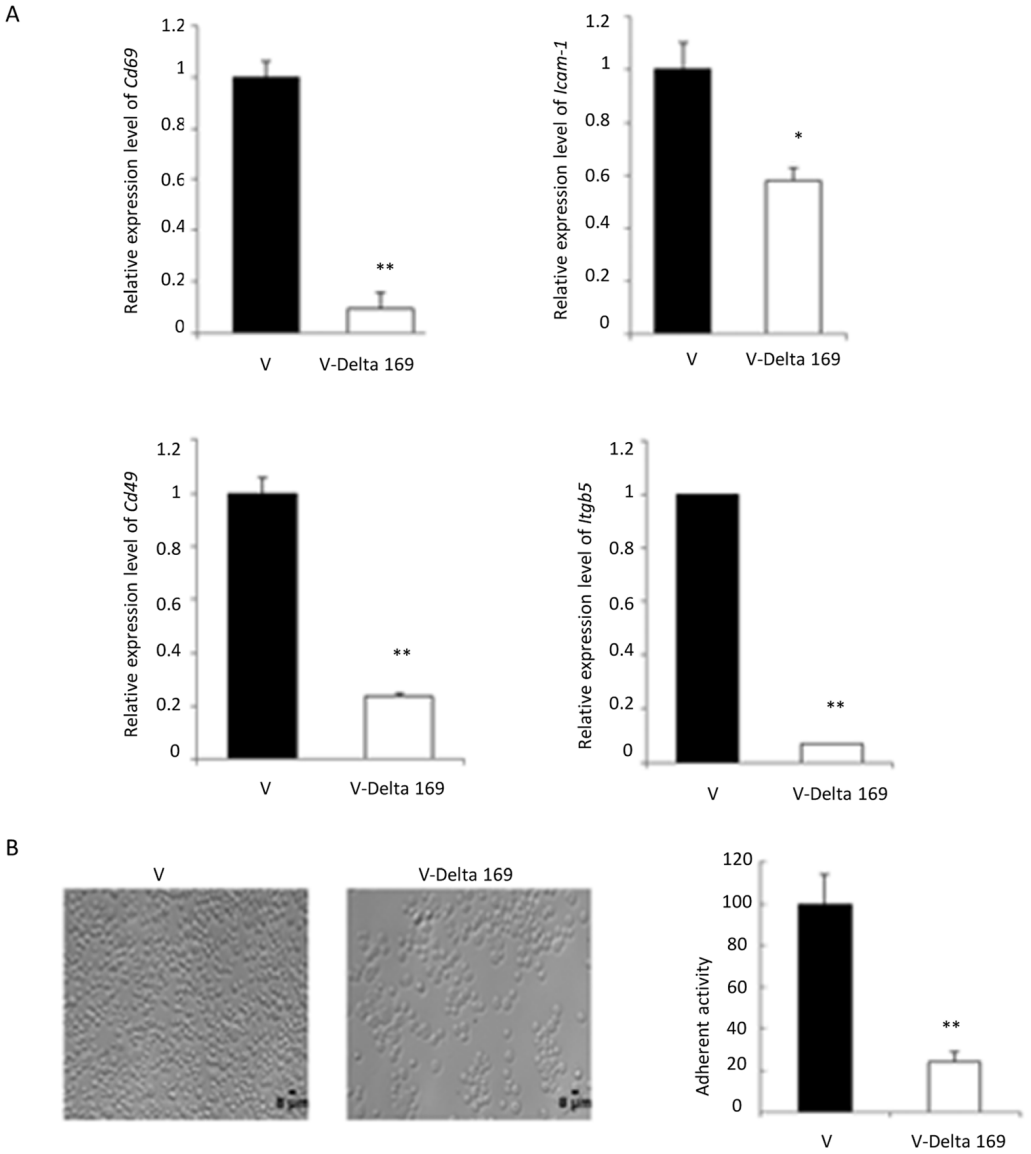
Role of c-Jun-target genes in infected macrophage adhesion to fibronectin. **A**, Infected macrophage adhesion is downregulated in the vaccine line (V-Delta 169) compared to virulent (V) Jed 4. RT-qPCR determined levels of genes (*cd69*, *icam-1*, *cd49* and *itgb5*) potentially involved in mediating adhesion. **B**, Virulent Jed 4 binds at high density to fibronectin compared to the V-Delta 169 vaccine line; 70% of fibronectin binding capacity is lost upon ablation of c-Jun.

Live attenuated vaccine lines lose adhesion [26], [28], and *mmp9* expression concomitant with loss of AP-1-activity [4]. Therefore, we examined the expression levels of a selection of AP-1-target genes *e.g. Cd69* [29], *Icam-1* [30], *Cd49* [31] and *Itgb5* [32]. The expression levels of these genes were significantly reduced in the engineered attenuated line V-Delta 169, and adhesion to fibronectin was 4-fold less than virulent (V: Jed4p18) control line (Figure 2). Significantly, ectopic expression of Flag-Δ169-c-Jun rapidly (within 3 weeks) dampened expression of *Cd69*, *Icam-1*, *Cd49 and Itgb5*, whereas it took 3-years of continuous culture (>100 pages) of the Ode vaccine line to achieve a similar attenuated phenotype [28].

### Inhibition of c-Jun results in reduced Matrigel traversal of the engineered attenuated line

**Figure 3:**
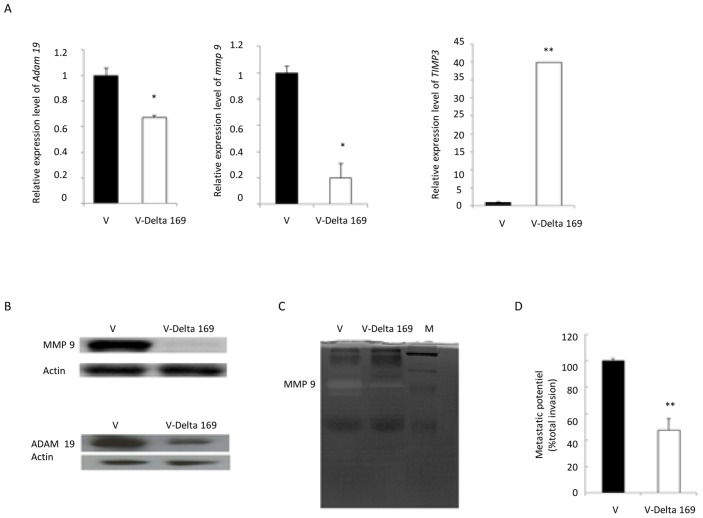
Inhibition of c-Jun results in reduced metastatic potential *in vitro*. **A**, RT-qPCR determination of relative expression shows *adam19* and *mmp9* are diminished and *timp3* levels are augmented in V-Delta 169 macrophages. **B**, MMP9 and ADAM19 protein levels are also decreased in V-Delta 169 macrophages. Amounts of actin were used as loading control. **C**, Gelatin zymography demonstrates that MMP9 proteinase activity is reduced in supernatants of V-Delta 169 macrophages. **D**, The invasive capacity, as determined by Boyden chamber assays show that V-Delta 169 macrophages have a 55% reduction in their ability to traverse Matrigel compared to virulent Jed 4 macrophages.

Upregulation of MMPs and reduced expression of their endogenous inhibitors TIMPs, by promoting cell migration and angiogenesis, contribute to tumour progression [33]. ADAM19 is highly expressed in human primary brain tumours, and expression and activity are associated with invasiveness [14]. Moreover, *Theileria* infection is known to induce AP-1-driven *mmp9* expression [2], [34] via induction of the methyltransferase SMYD3 that promotes trimethylation of histone H3K4 (H3K4me3) at the *mmp9* promoter [34]. Importantly, concomitant with loss of virulence MMP9 expression is dampened in the Ode vaccine line [4]. Our published microarray analyses of the Ode vaccine line indicated that 13 different *mmps*, *timps 1–4* and 48 *adams*-related genes are expressed by *T. annulata*-infected macrophages [24]. So, we confirmed that *mmp9*, *adam19* and *timp3* were expressed in virulent (V: Jed4p18) macrophages and that ectopic expression of Flag-Δ169-c-Jun in the engineered attenuated line altered their expression (Figure 3). *Theileria*-infection of Jed 4 macrophages (V: Jed4p18) induces both *adam19* and *mmp9* expression and appears to repress that of *timp3*. Conversely, ectopic expression of Flag-Δ169-c-Jun leads to a dampening of *adam19*, a reduction in *mmp9*, and de-repression of *timp3* expression. The protein levels of MMP9 and ADAM19 reflect alterations in amounts of their corresponding mRNA (Fig. 3B, left) and for MMP9 this is further reflected in loss of its gelatinase activity (Fig. 3B, centre). Consequently, the invasive capacity of the engineered attenuated line, as determined in Matrigel chamber assays, is significantly reduced (Fig. 3.D). These *in vitro* correlates of *Theileria*-infected macrophage virulence suggest that the engineered attenuated Jed 4 line would have a reduced capacity to disseminate *in vivo*.

### Dissemination of transformed Jed 4 macrophages in Rag2/γC mice

**Figure 4:**
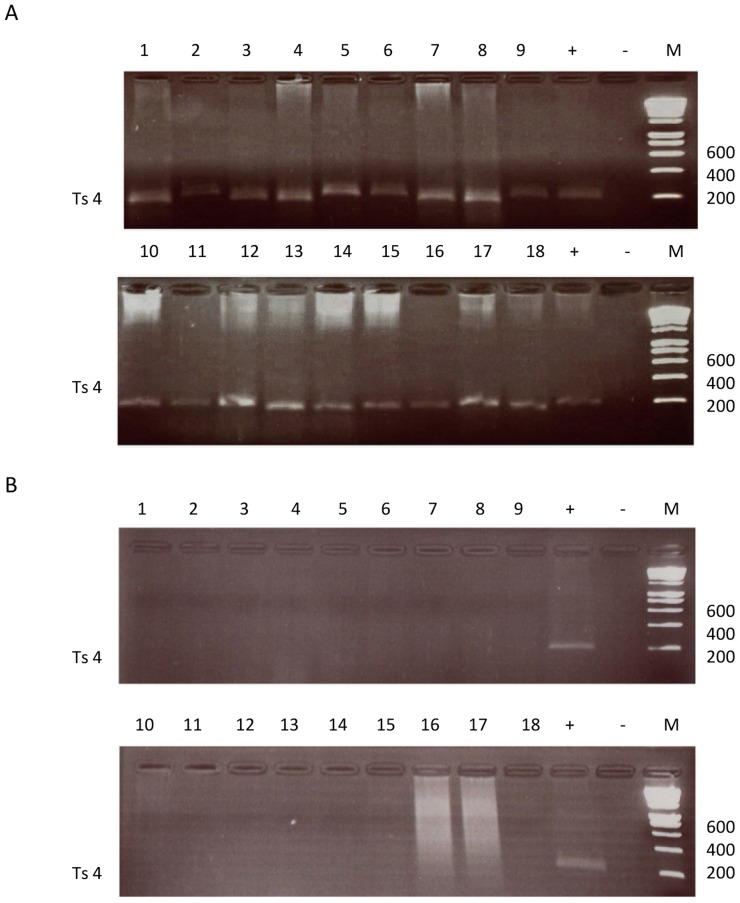
Dissemination of Jed 4 macrophages in Rag2/γC mice. **A (Virulent line) & B (Engineered attenuated line)**, The Ts4 micro-satellite locus was amplified from DNA extracted from the lungs (tracks 1–7), kidneys (tracks 8–11) and spleens (tracks 12–18) of 18 mice injected with virulent Jed 4 macrophages. Apart from a Ts4 specific band in the positive control no parasite DNA was amplified from extracts of lungs (tracks 1–8), kidneys (tracks 9–11) and spleens (tracks 12–18) of 18 mice injected with V-Delta 169 macrophages. (+) = Positive control DNA, (−) = negative control water, MW = molecular-weight size marker (200 bp ladder).

It is likely that the reduced dissemination of live attenuated vaccines, as estimated in heterologous mouse models, allows them to confer protection of cattle against theileriosis without killing the vaccinated animal [35]. Moreover, reduced dissemination of live attenuated vaccines has been ascribed to loss of MMP9 activity [36]. Above, we showed that the engineered attenuated Jed 4 line has not only reduced MMP9 activity, increased *timp3* levels and ablated ADAM19 expression compared to control virulent Jed 4 (V: Jed4p18) macrophages, so we tested whether this translated into a failure to disseminate by their subcutaneous injection into Rag2/γC mice [12]. Dissemination of virulent *Theileria*-transformed Jed 4 macrophages (V: Jed4p18) gave rise to metastases in kidneys, spleen and lungs, and the presence of *T. annulata* in these tumours was confirmed by PCR detection of 4 different microsatellite parasite markers (Ts4, Ts6, Ts8 and Ts25) in tissue extracts of the 3 different organs and shown in Figure 4 is the result with Ts4. In contrast, we failed to detect tumours (or parasite DNA) in the same organs of mice injected with the engineered attenuated line. Thus, in this heterologous mouse model for *Theileria*-transformed macrophage dissemination ectopic expression of Flag-Δ169-c-Jun that diminishes MMP9/ADAM19 and increases TIMP3 expression led to ablation of tumour dissemination by the engineered attenuated line.

## Discussion

Vaccination trials with recombinant *T. annulata* antigens have shown promise, but currently they are not in field use [37], [38]. Due to the emergence of parasite resistance to buparvaquone (Bw720c) [39] live attenuated vaccines thus remain an important control measure for tropical theileriosis [40]. However, the immunity conferred by attenuated vaccines against heterologous challenge decreases significantly with the time in culture taken to attenuate them [19]. One reason is the infected macrophage becomes less immunogenic with the length of time in culture, combined sometimes with selection of less virulent intracellular parasites better adapted for growth *in vitro* [3]. Here, we have attempted to circumvent the long-term culture via the rapid (3-week) ablation of c-Jun in virulent Jed 4 (V-Delta 169) infected macrophages. Injection of the engineered line into Rag2/γC mice demonstrated it was completely attenuated for dissemination. Parasites were only detected by PCR amplification of 4 micro-satellites (Ts4, Ts6, Ts8 and Ts25) in 3 different organs of mice injected with virulent Jed4 (V: Jed4p18) and Figure 4 shows the result for the Ts4 locus. Loss of dissemination could not be ascribed to rapid selection of a less virulent parasite within V-Delta 169 macrophages. This underscores that a key determinant of *Theileria*-infected macrophage dissemination is upregulation of c-Jun, rather than *stricto senso* the presence of a virulent intracellular parasite.

The long-term culture traditionally required to attenuate macrophage virulence is believed to sometimes generate either the selection of an attenuated parasite, or the selection of a less virulent parasite subpopulation present in the original isolate [3]. In this scenario, if upon vaccination attenuated parasites become transferred to endogenous macrophages the ensuing infection would be itself avirulent. Thus, one might have poor immunity generated by the live attenuated vaccine, but the resulting post-vaccination “carrier state” would also be attenuated [41]. As parasites in the rapidly generated V-Delta 169 engineered line have the same micro-satellite profile as virulent as Jed 4 it implies that if transferred to endogenous macrophages in vaccinated animals they could eventually be taken up during a subsequent tick blood meal with a risk of transfer of virulent parasites to non-vaccinated animals. Potential transfer of virulent parasites from V-Delta 169 macrophages to endogenous macrophages can't be tested in Rag2/γC mice, as *T. annulata* only grows in bovine leukocytes. These studies therefore, will be performed in the future by experimental vaccination of calves at the National Veterinary School in Tunisia. Experimental vaccination with macrophages genetically engineered for ablation of a specific host cell virulence trait should lead to a better appreciation of how transformed macrophage dissemination contributes to tropical theilriosis disease pathology.
